# Disease burden, clinical management and unmet treatment need of patients with moderate to severe atopic dermatitis; consensus statements, insights and practices from CERTADE (Central/Eastern EU, Russia, Turkiye AD Experts) Delphi panel

**DOI:** 10.3389/fmed.2024.1402493

**Published:** 2024-06-19

**Authors:** Magdalena Trzeciak, Lidia Rudnicka, Petr Arenberger, Burhan Engin, Andrey L'vov, Sibel Alper, Erkan Alpsoy, Nina Benáková, Svetlana Bobko, Murat Borlu, Magdalena Czarnecka-Operacz, Olga Elisyutina, Tulin Ergun, Ilgen Ertam, Elena Fedenko, Olga Filipovská, Daria Fomina, Aida Gadzhigoroeva, Martina Kojanová, Aleksandra Lesiak, Anna Michenko, Nikolay Murashkin, Witold Owczarek, Esen Özkaya, Zuzana Plzáková, Adam Reich, Marie Selerova, Burcu A. Gurbuz

**Affiliations:** ^1^Department of Dermatology, Venereology and Allergology, Medical University of Gdańsk, Gdańsk, Poland; ^2^Department of Dermatology, Medical University of Warsaw, Warsaw, Poland; ^3^Department of Dermatovenerology, Third Faculty of Medicine, Charles University of Prague, Prague, Czechia; ^4^Department of Dermatology and Venerology, Cerrahpaşa Medical Faculty, Istanbul University-Cerrahpaşa, İstanbul, Türkiye; ^5^Department of Dermatology, Federal State Budgetary Institution of Continuing Professional Education “Central State Medical Academy”, Moscow, Russia; ^6^Department of Dermatology, Medical Research and Educational Center, Lomonosov Moscow State University, Moscow, Russia; ^7^Department of Dermatology and Venereology, Koç University, İstanbul, Türkiye; ^8^Department of Dermatology and Venereology, Akdeniz University, Antalya, Türkiye; ^9^Department of Dermatovenereology, 1st Medical Faculty, Charles University, Prague, Czechia; ^10^Moscow Scientific and Practical Centre of Dermatovenereology and Cosmetology, Moscow, Russia; ^11^Department of Dermatology and Venereology, Erciyes University, Kayseri, Türkiye; ^12^Department of Dermatology, Medical University of Poznań, Poznań, Poland; ^13^NRC Institute of Immunology FMBA of Russia, Moscow, Russia; ^14^Department of Dermatology, Marmara University, İstanbul, Türkiye; ^15^Department of Dermatology, Ege University, İzmir, Türkiye; ^16^Department of Dermatology, Hospital in Ústí nad Labem, Ústí nad Labem, Czechia; ^17^Centre of Allergy and Immunology, Clinical State Hospital 52, Moscow Ministry of Healthcare, Moscow, Russia; ^18^Department of Dermatology, I. M. Sechenov Moscow Medical University, Moscow, Russia; ^19^Dermatology and Venereology Clinic, Medical University, Łodź, Poland; ^20^Department of Dermatology, Federal State Autonomous Institution, Scientific Centre of Children's Health of the Ministry of Health of the Russian Federation, Moscow, Russia; ^21^Department of Dermatology, Military Institute of Medicine, Warsaw, Poland; ^22^Istanbul Faculty of Medicine, Department of Dermatology and Venereology, Istanbul University, İstanbul, Türkiye; ^23^Department of Dermatology, University of Rzeszow, Rzeszów, Poland; ^24^Department of Dermatology, AGEL, Prague, Czechia; ^25^Pfizer, İstanbul, Türkiye

**Keywords:** atopic dermatitis, disease burden, management, treatment, moderate-to-severe AD

## Abstract

**Background:**

There is limited insight into the current disease burden and everyday clinical management of moderate-to- severe AD in Poland, Czechia, Russia, and Turkiye. Therefore, this study aimed to get information-driven insights regarding the current disease burden and clinical management of patients with moderate-to-severe AD with common and differentiating aspects of the patient journey and establish a consensus.

**Methods:**

In this modified 2-round Delphi panel, 133 questions were asked in total to 27 dermatologists. A consensus was achieved when 70% of the panel members strongly agreed or agreed (or strongly disagreed or disagreed) with an item. Statements with <40% agreement dropped from the Delphi rounds and were not repeated.

**Results:**

The results state that AD has a significant impact on the quality of life for both patients and their families with social and economic consequences in these countries. While there were significant dissimilarities regarding the current treatment approach by preference order and treatment duration among participants, there was also a high percentage of consensus on literature and guideline-based statements. Current topical therapies and the immune response modifiers were not found to be sufficient by panelists to cover the therapeutic needs of patients with moderate-to-severe AD. Moreover, panelists highlighted the significant burden of adverse events with the off-label use of currently available immunosuppressants.

**Conclusions:**

These results underlined that there is a significant disease burden with an unmet treatment need for patients with moderate-to-severe AD in Poland, Czechia, Russia, and Turkiye.

## Introduction

Atopic dermatitis (AD) is one of the most common inflammatory skin conditions in both children and adults either as a persistent disease from childhood or a recurring or adult-onset. AD has a lifetime prevalence of well over 20% in many affluent country settings with a substantial variation. AD affects up to 25% of the pediatric population and up to 5% of adults worldwide (1–13). However, epidemiological studies on childhood and adulthood AD in different continents are still lacking.

Although AD is not a fatal disease, severity and intrusiveness of itch, sleep disturbances and localization of skin lesions to certain anatomic distributions may have a substantial impact on the quality of life for both patients and their families (14). Clinical presentation and severity of AD vary widely based on patient factors such as age, skin type, ethnicity, and other comorbidities. AD diagnosis and severity assessment are not always straightforward since there are no definitive diagnostic tests, no reliable laboratory tests, or biomarkers to assess the severity of AD (15–17). As a result, clinicians must rely upon clinical assessments of disease parameters, which can be subjective and difficult to standardize.

Data suggests that extrinsic environmental factors, intrinsic immune mechanisms and genetic factors play an important role in AD disease progression (18). With such a complex etiology and heterogenic patient population, available therapies only provide symptomatic control rather than a cure for AD patients (18, 19). Although many patients with AD can achieve disease control, previously available conventional treatments often have inadequate efficacy in patients with moderate-to-severe AD (20). Until recently, there were few approved systemic treatment options for patients with moderate-to-severe AD in many countries which are often limited by contraindications, side effects and commonly associated with disease rebound (20). Instead, clinicians choose from several off-label immunosuppressants, which may have serious adverse effects. As a result, a significant number of these patients remain untreated (20, 21). This unmet treatment need in AD poses a substantial burden on healthcare resources with considerable cost implications. Beyond its economic cost, atopic dermatitis bears a significant burden to society. The Global Burden of Diseases Study demonstrated that skin diseases were the fourth leading cause of non-fatal disease burden (22).

Disease management in AD also varies significantly across the countries because both clinical guidance and economic factors are taken into consideration during clinical decision-making. Consequently, there is limited insight into the current disease burden and everyday clinical management of moderate-to- severe AD in Poland, Czechia, Russia, and Turkiye. Therefore, this study aimed to get information-driven insights from Poland, Czechia, Russia, and Turkiye regarding the current disease burden and clinical management of patients with moderate-to-severe AD with common and differentiating aspects of the patient journey and establish a consensus.

## Methods

### Study design

This modified Delphi study was consisted of two rounds and conducted over a 3-month period (between 26/11/2020 and 08/04/2021) to understand the current disease burden, diagnostic approach, treatment preferences, and treatment response assessment to identify unmet medical needs in moderate to severe AD from physician perspectives in Poland, Czechia, Russia, and Turkiye.

The Delphi method is a well-established approach to reach consensus and achieve mutual decisions among experts on a number of issues when there is only limited data or scarce body of information available in relevant fields (23). There are no set guidelines or recommendations for deciding on the appropriate number of Delphi participants as this is likely to change depending on the purpose of the Delphi survey. However, a minimum sample of 15–20 participants is said to be adequate if a homogeneous group of participants is targeted. Briefly, the method involves a facilitator sending a questionnaire to the expert participants and analyzing their answers anonymously. Then this facilitator uses these answers to develop a new questionnaire and the cycle is repeated (24). Usually, the classic Delphi method comprises three or more rounds, whereas the modified versions can be finalized by two rounds. After each round experts are encouraged to revise their earlier answers in light of the replies of other members (25). The modification with fewer rounds allows researchers to avoid losses of acceptable response rates and the negative influence on the panelists' interest due to prolonged duration of the process (26).

In the present study, a modified Delphi method was used to reach consensus. The questions were designed using either a 5-point Likert response scale or multiple-choice answers with an additional open-ended choice. A total of 133 questions were asked in total covering disease burden, diagnosis, disease severity, treatment, treatment response, treatment landscape, and unmet medical need.

**Figure 1:**
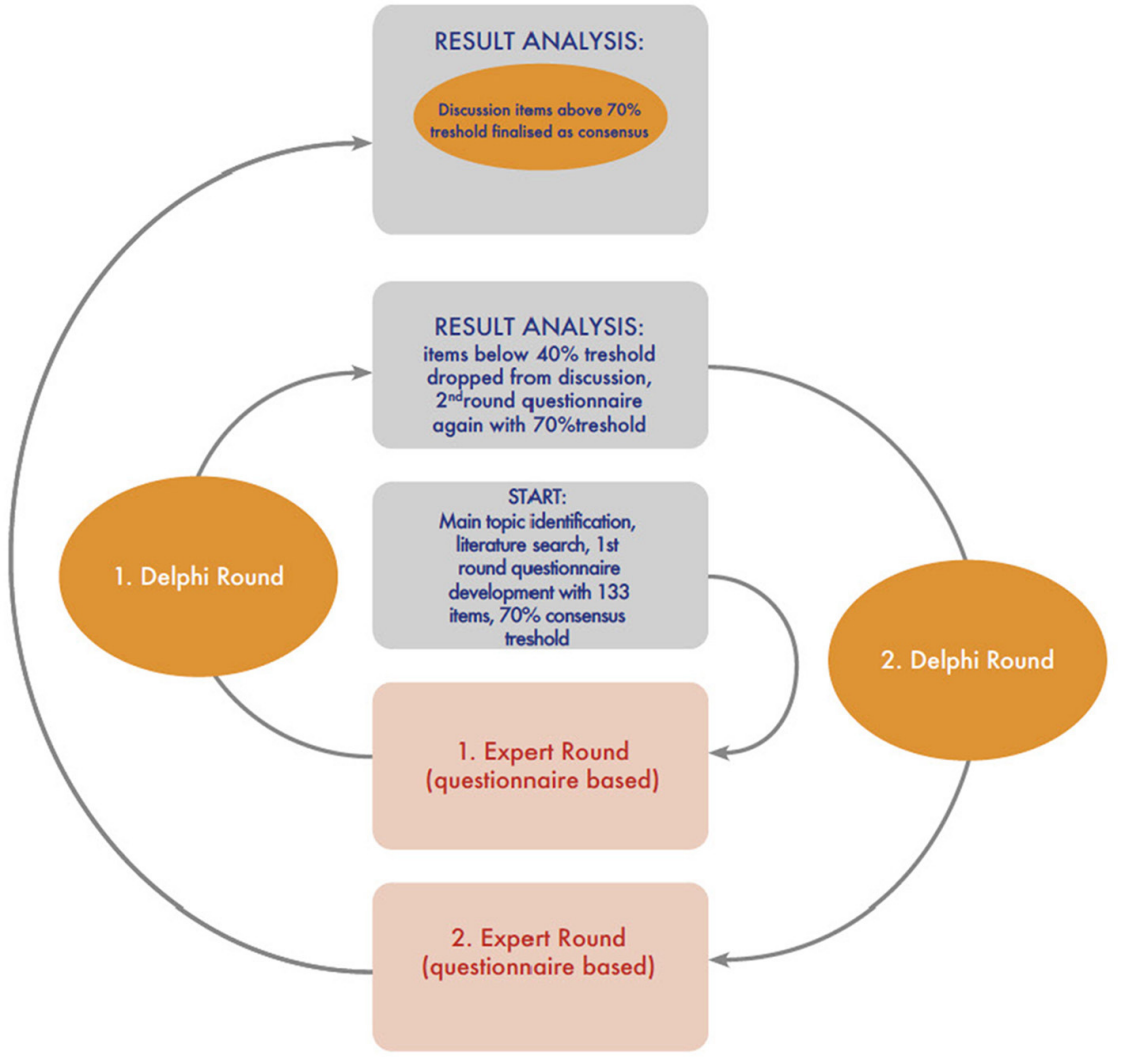
Study design and workflow.

Three separate electronic questionnaires were used to collect the respondents' opinions on the first round of this study due to the comprehensiveness of the subjects. A consensus/dominant approach was achieved when 70% of the panel members strongly agreed or agreed (or strongly disagreed or disagreed) with a statement or selected the same answer. Statements with <40% agreement dropped from the Delphi rounds and were not repeated. The questions asked during the second round of this study were repeated either by using the same question or a rephrased content based on the commentaries/corrections made by the participants during the first round. Contradictions between different countries were accepted as a non-consensus factor. The study design is summarized in Figure 1.

### Participants and process

At the beginning of the project, a scientific committee formed by four dermatologists; one study coordinator and three committee members from Czechia (1), Poland (1; as study coordinator), Turkiye (1), and Russia (1) with proven experience in AD was established. Each scientific committee member is either a board member of an academic association, contributed to guideline developments on this subject or published articles on AD. The study coordinator supervised the design and progression of the study including data analysis. The main topics to be investigated in the study questionnaire were brought together by an independent expert consultant (facilitator) and then selected and approved by the scientific committee members based on the limited world evidence. Questions were then prepared by the same independent expert consultant in order to avoid the scientific committee's participation bias. Candidates to participate in the study were chosen according to their specific interest and extensive experience in AD. Selected participants were specialists in Dermatology (25) and Allergology (2) working in university and public hospitals in Russia (8), Turkiye (7), Poland (6), and Czechia (6) with 10 or more years of experience with AD. Each panelist is also either a board member of an academic association, contributed to guideline developments on this subject or published articles on AD. Overall, 14 of the experts were involved in the final manuscript validation.

Publishing period of the study delayed for nearly 2 years due to some of the participants' serious health problems and several other logistic issues. Literature review is revisted and utilized accordingly within the Discussion Section.

## Results

### Questionnaire structure

The study questionnaire was consisted of 5 different sections covering disease burden, diagnosis, disease severity, treatment, treatment response and unmet treatment need topics. Each section included 67 statements and 66 questions aiming to understand the general perception and acceptance of the available information as well as to investigate the current status of the care of patients with AD in these countries.

### General results

Each participant answered all questions. The consensus was achieved on 50 of the 67 statement questions. The percentages of consensus ranged between 57% and 100%. The lowest consensus percentage was in the treatment section and the highest percentages in the Disease burden and Disease severity Sections.

Of the other 66 questions, the most common observations, perspectives and practices were again in the disease severity and disease burden sections where the clinical practices were observed as significantly diverse between countries and even mostly varied within in each country regarding the treatment response assessment and treatment preferences (Table 1).

**Table 1:** General results.

**Statement sections**	**Total statement numbers in each section**	Consensus in each section *n* (%)^*^
A. Disease burden	10	10 (100)
B. Diagnosis	8	7 (87)
C. Disease severity	5	5 (100)
D. Treatment	28	16 (57)
E. Treatment response and unmet need	16	12 (75)
**Common observations, perspectives and practices** ^*^		
**Survey Sections**	**Questions asked in each section**	**Common perspectives and practice** ^*^
1. Disease burden	10	7 (70)
2. Diagnosis	20	9 (45)
3. Disease severity	6	5 (83)
4. Treatment	19	7 (36)
5. Treatment response and unmet need	11	3 (11)

^*^More than 70% of the total participants and of the participants in each country.

### Disease burden

The consensus was achieved in all the statement items in this section. The highest consensus was for “Lesions in visible areas impair patient's quality of life to a greater extent” statement. Participants agreed on the fact that moderate-to-severe AD causes significant psychological disorders (anxiety, mood disorders) and results in a dramatic impact on quality of life for both patients and their families (96.3%). They also reported AD as an impairing and a disabling disease (92.6% and 81.5% respectively) which causes significant work and productivity impairment in adults with moderate-to-severe condition (92.6%).

The common observations provided by the participants were as follows; nearly half of moderate to severe AD patients report that their condition has major consequences (career, work productivity, social relationships, and financial burden) on their lives (96.3%), quality of life indexes are not used as much as they should to evaluate AD patients in daily practice (96.3) and AD related impairments and disabilities are not adequately publicized/emphasized enough in media to raise public awareness on AD disease burden (92.5%) (Table 2).

**Table 2:** Results regarding AD disease burden perception.

Statements^*^	**Consensus %**
**Disease burden**	
Moderate-to-severe AD causes significant psychological disorders (anxiety, mood disorders) and results in a dramatic impact on the quality of life for both patients and their families.	96.3
Clinical signs such as periorbital darkening, Dennie-Morgan lines and the “dirty neck” appearance cause stigmatization of moderate-to-severe AD patients.	77.7
Lesions in visible areas impair patient's quality of life to a greater extent.	100
Tendency to suicide is higher in patients with moderate-to-severe AD than general population without AD.	85.2
Moderate-to-severe AD causes significant work and productivity impairment in adults.	92.6
AD is an impairing disease.	92.6
AD is a disabling disease.	81.5
**Common observations, perspectives and practices** ^*^	**%**
**Disease burden**	
The rate of referring to a healthcare provider is increased in AD patients compared to the general population without AD.	85.1
The rate of emergency room visits is higher in AD patients whose symptoms are not under adequate control when compared with AD patients under control.	88.8
The rate of visits to other healthcare providers apart from emergency room is higher in AD patients whose symptoms are not under adequate control when compared with AD patients under control.	81.4
Nearly half of moderate to severe AD patients report that their condition has major consequences (career, work productivity, social relationships, financial burden) on their lives.	96.3
AD is perceived as an impairing disease in our society.	74
AD related impairments and disabilities are not adequately publicized/emphasized enough in media in order to raise public awareness on AD disease burden.	92,5
**Clinical practice**	
I take into consideration the patient's quality of life when making treatment decisions.	88.8
Quality of life indexes are not used as much as they should to evaluate AD patients in daily practice in my country.	96.3
Psychiatric consultation for patients with moderate to severe AD are not performed much as they should to evaluate AD patients in daily practice in my country.	70.3

^*^More than 70% of the total participants and of the participants in each country. AD, atopic dermatitis.

### Diagnosis and patient journey

The consensus was achieved in 7 of the 8 statement items in this section. Of the 7 items, the consensus with the highest percentages were “In certain situations, skin biopsies should be considered to exclude other conditions (such as early-stage T-cell cutaneous lymphoma, psoriasis, or dermatitis herpetiformis)” (96.3%), “Currently, there are no validated biomarkers that aid in the diagnosis of AD” (88.8%) and “Diagnostic criteria systems are sufficient to diagnose AD” (85.2%).

Regarding the common observations, the speciality, adult patients with AD most commonly refer to with their first symptoms was chosen as Dermatology (96.3%), however experts from Russia also chose Allergy-Immunology as equally referred. Additionally, the speciality, children with AD (families) most commonly refer to with their first symptoms was chosen as Pediatrics (81.4%), where experts from Poland and Czechia chose Dermatology as the most referred. Both child and adult patients with AD were reported to have their first diagnosis most commonly in dermatology in all countries (88.8%, 100%). The average time for AD patients to reach a diagnosis from their first symptom varied among experts' perspective, however they reported it as <6 months (74.2%) (Czechia and Turkiye <4 months, Russia <1 month) (Table 3).

**Table 3:** Results regarding AD diagnostic approach and patient journey.

Statements^*^	**Consensus %**
**Diagnosis**	
AD requires a multidisciplinary (such as pediatrics, dermatology, and immunology) approach regarding the diagnosis of the condition.	70.3
Diagnostic criteria systems are sufficient to diagnose AD.	85.2
In certain situations, skin biopsies should be considered to exclude other conditions (such as early-stage T-cell cutaneous lymphoma, psoriasis, or dermatitis herpetiformis).	96.3
Rationales and criteria of performing a biopsy for AD diagnosis is not well established.	81.4
IgE, allergen specific IgE and the presence of eosinophilia provide differentiation between intrinsic and extrinsic AD	74
Rationales and criteria of performing a prick test for AD diagnosis is not well established	85.1
Currently, there are no validated biomarkers that aid in the diagnosis of AD	88.8
**Common observations, perspectives and practices** ^*^	**%**
**Diagnostic journey**	
The specialty/ies, children with AD (families) most commonly refer to with their first symptoms: Pediatrics^**^(Experts from Poland and Czechia chose Dermatology as the most referred)	81.4
The specialty/ies, children with AD (families) most commonly refer to with their first symptoms: Dermatology^**^(Experts from Russia chose Allergy-Immunology as the second most common)	74
The specialty/ies, adult patients with AD most commonly refer to with their first symptoms: Dermatology^**^(Experts from Russia chose Dermatology and Allergy-Immunology as equally as referred)	96.3
The specialty/ies, child patients with AD most commonly have their diagnosis at: Dermatology	88.89
The specialty/ies, adult patients with AD most commonly have their diagnosis at: Dermatology	100
The average time for AD patients to reach a diagnosis from their first symptom: <6 months^**^ (Czechia and Turkiye <4 months, Russia <1 month)	74.2
**Clinical practice**	
I use a diagnostic criteria system to diagnose AD patients in my daily practice.	88.9
The diagnostic criteria system/s I prefer in order to diagnose AD patients in my daily practice: Hanifin and Rajka criteria.	77.8
I check IgE, allergen specific IgE and the presence of eosinophilia for the differential diagnosis of intrinsic and extrinsic AD in children ^**^(Poland has various approaches within).	77.7

^*^More than 70% of the total participants and of the participants in each country. ^**^Variation between countries, total response is still over 70%. AD, atopic dermatitis; IgE, immunoglobulin E.

### Disease severity

Of the five statement items, the consensus was reached in all of them with the highest percentages regarding that “AD patients who have significantly impaired quality of life can be considered to have moderate-to-severe AD regardless of BSA” (96.3%) and the statement that “AD patients with minimum involvement of 10% BSA may be considered as moderate-to-severe AD” (92.6%). Furthermore, the scoring system/s preferred when estimating the severity of disease, SCORAD was chosen as the most common (74%) (Experts from Czechia chose EASI as the most common, IGA-BSA and SCORAD the least common) and EASI as the second most common among all countries (66.6%). However, disease severity scale/scoring systems were reported as not being used as much as they should to evaluate AD patients in daily practice (88.8%) (Table 4).

**Table 4:** Results regarding AD disease severity perception and assessment.

**Statements**	**Consensus %**
**Disease severity**	
AD patients with a minimum involvement of 10% BSA may be considered as moderate-to-severe AD.	92.6
AD patients who have individual lesions with moderate-to-severe features may be considered as moderate-to-severe AD regardless of BSA	74.2
Involvement of highly visible areas or those important for function (e.g., neck, face, genitals, palms and/or soles) in AD patients may be considered as moderate-to-severe AD regardless of BSA	85.1
AD patients who have significantly impaired quality of life can be considered to have moderate-to-severe AD regardless of BSA	96.3
Using systems that allow patients' self-assessment of disease severity, provides an ideal treatment approach by combining the patient's and physician's perspective in AD management	91.5
**Common observations, perspectives and practices** ^*^	**%**
**Clinical practice**	
Disease severity scale/scoring systems are not used as much as they should to evaluate AD patients in daily practice in my country	88.8
I use disease severity scoring systems to assess treatment response in AD	74
The scoring system/s I prefer when estimating the severity of disease: SCORAD^**^(Experts from Czechia chose EASI as the most common, IGA-BSA and SCORAD the least common), EASI the second most common among all countries (66.6%)	74
I need a more practical scoring system (ex: less time consuming, more inclusive of other factors rather than just BSA percentage) to assess severity of disease. ^***^	74.2

^*^More than 70% of the total participants and of the participants in each country. ^**^Variation between countries, total response is still over 70%. ^***^Czechia was below 70% regarding this need. AD, atopic dermatitis; BSA, body surface area; SCORAD, scoring atopic dermatitis; EASI, eczema area and severity index; IGA-BSA, Investigator Global Assessment and body surface area.

### Treatment

The lowest consensus percentage was in the treatment section. There were significant dissimilarities among participants, even within the same country, when they were asked to provide their current treatment approach by preference order and treatment duration. However, they agreed on some literature based statements such as; “Targeted patient education and support is essential for each step of AD treatment” (96.2%), “Proactive treatment (twice a week for 2 consecutive days to previously affected areas of skin for an undefined but long-term period) with TCS or TCIs often prevents disease flare-up” (92.6%), “Phototherapy should be tried in adults with persistent moderate to severe AD despite optimal topical therapy, before considering systemic agents”(92,6%), “If treatment responses to topical treatments are inadequate, systemic therapies can be initiated in children over 12 years of age and in adult patients with moderate to severe AD” (both 96.3%), Dupilumab can be initiated as a first-line systemic treatment in children over 12 years of age and in adults with moderate-to-severe AD in whom the disease could not be controlled with optimal topical and/or phototherapies (From a scientific perspective assuming biologic treatment is available and reimbursed for this condition for your patients) (85.2%), efficient systemic treatment started at an early stage may prevent the development of disease-specific comorbidities (85.1%).

Participants stated that clinical response, duration of remission and side effects were the most important factors which affected their treatment preference in AD.

Although the most referred guideline to manage AD patients was reported as EADV in general (70.3%), participants from Poland and Russia stated that they used local guidelines more than EADV (27). Seventy seven percent of the participants reported AD as an under-treated disease in their country and 70% stated that the collaboration between specialties needed significant improvement in their country to diagnose and treat AD patients without delays (Table 5).

**Table 5:** Results regarding treatment preferences and management.

**Item**	**Consensus %**
**Treatment**	
AD requires a multidisciplinary approach regarding treatment management.	77.7
The primary goal of the treatment in a patient diagnosed with AD is to achieve a cure.	74
In the event that curative treatment is impossible, stabilizing dermatitis at the lowest level is a primary goal.	100
In the event that curative treatment is impossible, reducing the symptoms of greatest concern to the patient is also a primary goal of the AD treatment (e.g., preventing infections, improving QoL, reducing itching and improving sleep)	100
Targeted patient education and support is essential for each step of AD treatment.	96.2
TCS treatments should be tried before moving on to other local and systemic treatments regardless of age.	70
Proactive treatment (twice a week for 2 consecutive days to previously affected areas of skin for an undefined but long-term period) with TCS or TCIs often prevents disease flare-up.	92.6
First- and second-generation antihistamines are not effective for use as a systemic treatment of AD.	74
Phototherapy should be tried in child patients over 12 years of age with persistent moderate to severe AD despite optimal topical therapy, before considering systemic agents.	70.3
Phototherapy should be tried in adults with persistent moderate to severe AD despite optimal topical therapy, before considering systemic agents.	92.6
If treatment responses to topical treatments are inadequate, systemic therapies can be initiated in children over 12 years of age with moderate to severe AD	96.3
If treatment responses to topical treatments are inadequate, systemic therapies can be initiated in adult patients with moderate to severe AD	96.3
Dupilumab can be initiated as a first-line systemic treatment in children over 12 years of age with moderate-to-severe AD in whom the disease could not be controlled with optimal topical and/or phototherapies (From a scientific perspective assuming biologic treatment is available and reimbursed for this condition for your patients.)	85.2
Dupilumab can be initiated as a first-line systemic treatment in adults with moderate-to-severe AD in whom the disease could not be controlled with optimal topical and/or phototherapies (From a scientific perspective assuming biologic treatment is available and reimbursed for this condition for your patients).	85.2
SCS, Dupulimab, CyC are suitable systemic treatments for children patients above 12 years of age with moderate to severe AD, depending on patient profiles they can be used as alternatives to each other if one of them doesn't provide adequate response.	74
Efficient systemic treatment started at an early stage may prevent the development of disease-specific comorbidities.	85.1
**Common observations, perspectives and practices** ^*^	**%**
**Patient journey insight items from different country settings**	
The specialty/ies, most commonly provide/s long-term follow-up for child patients with AD (mild an moderate-severe didn't vary): Dermatology (second most common referral unit for moderate to severe AD, Allergy-Immnulogy: 51.8%).	96.3
The specialty/ies, most commonly provide/s long-term follow-up for adult patients with AD (mild an moderate-severe didn't vary): Dermatology (second most common referral unit for moderate to severe AD, Allergy-Immnulogy: 51.8%).	100
AD is an under-treated disease in my country.	77.7
The collaboration between specialties needs significant improvement in my country to diagnose and treat AD patients without delays.	70.3
**Clinical practice insight items from different country settings**	
The treatment guidelines I follow to treat my AD patients: EADV^**^(Poland and Russia use local guidelines the more than EADV).	70.3
SCS or CyC are the first line systemic treatments that I generally use for adult patients with moderate-severe AD despite optimal local therapy^**^(Poland and Check Rep above 80%, Turkiye and Russia have different approaches within the country).	74
Clinical response, duration of remission and side effects are the most important factors which affect my treatment preference in AD.	99.9

^*^More than 70% of the total participants and of the participants in each country chose the same answer. ^**^Variation between countries, total response is still over 70%. AD, atopic dermatitis; EADV, European Academy of Dermatology and Venereology; SCS, Systemic Corticosteroids; CyC, Cyclosporine.

### Treatment response and unmet need

Panelists defined treatment failure as an inadequate clinical improvement despite appropriate dose and duration of and adherence to a therapeutic agent with full consensus (100%). They also agreed on other treatment failure definitions such as; failure to achieve stable long-term disease, presence of ongoing impairment (e.g., pruritus, pain, loss of sleep, and poor quality of life) or unacceptable adverse events or poor tolerability experienced with the treatment despite appropriate dose and duration of and adherence to a therapeutic agent (92.6%, 88.8%, and 81.4%). Panelists stated that response to treatment in AD patients should be assessed by disease severity scores and they agreed on the fact that there are no generally accepted criteria for defining treatment failure in AD patients (96.3% and 92.6%).

Current topical therapies and the immune response modifiers were not found to be sufficient by panelists to cover the therapeutic needs of patients with moderate-to-severe AD (92.6%). Moreover, consensus was reached regarding that there is a significant burden of adverse events with off-label use of currently available immunosuppressants (85.2%) and there is a significant unmet need for novel topical and systemic agents that offer prolonged remission and a safe side-effect profile in long-term moderate to severe AD treatment (both 100%) (Table 6).

**Table 6:** Results regarding treatment response and unmet need perceptions and observations.

**Statements**	**Consensus %**
**Treatment response**	
Inadequate clinical improvement despite appropriate dose and duration of and adherence to a therapeutic agent can be defined as treatment failure.	100
Failure to achieve stable long-term disease control despite appropriate dose and duration of and adherence to a therapeutic agent can be defined as treatment failure	92.6
Presence of ongoing impairment (eg, pruritus, pain, loss of sleep, and poor quality of life) while on treatment despite appropriate dose and duration of and adherence to a therapeutic agent can be defined as treatment failure.	88.8
Unacceptable adverse events or poor tolerability experienced with the treatment despite appropriate dose and duration of and adherence to a therapeutic agent can be defined as treatment failure.	81.4
Response to treatment in AD patients should be assessed by disease severity scores (such as SCORAD, EASI, IGA etc.)	96.3
There are no generally accepted criteria for defining treatment failure in AD patients.	92.6
**Unmet need**	
Topical therapies do not sufficiently cover the therapeutic needs of patients with moderate-to-severe AD.	92.6
The current immune response modifiers do not sufficiently cover the therapeutic needs of patients with moderate-to-severe AD.	92.6
There is a significant burden of adverse events with off-label use of currently available immunosuppressants	85.2
There is a significant unmet need for novel topical agents that offer prolonged remission and a safe side-effect profile in long-term moderate to severe AD treatment.	100
There is a significant unmet need for novel systemic agents that offer prolonged remission and a safe side-effect profile in long-term moderate to severe AD treatment.	100
**Common observations, perspectives and practices** ^*^	**%**
**Clinical practice**	
I use disease severity scoring systems to assess flares in AD.	70.3
**Observations**	
More than half of the children AD patients' families have topical steroid phobia.	74
Families of child AD patients have more fears over systemic steroids' side effects than topical steroids	85

^*^More than 70% of the total participants and of the participants in each country chose the same answer. SCORAD, scoring atopic dermatitis; AD, atopic dermatitis; EASI, eczema area and severity index; IGA, Investigator Global Assessment.

## Discussion

The Delphi method consensus study on atopic dermatitis (AD) has brought to light several significant findings that resonate with the broader academic discourse on the subject. A paramount consensus was reached on the notion that lesions in visible areas profoundly impair a patient's quality of life. Such lesions can lead to heightened self-consciousness, social stigmatization, and emotional distress. A study by Lio et al. specifically highlighted that AD lesions in visible areas, including the head and neck, hands, and fingers, were most bothersome to patients, with a significant association to various Dermatology Life Quality Index (DLQI) domains (28). These visible lesions were also linked to increased symptoms of anxiety and depression, emphasizing the profound psychological toll they can take (29). Another study by Ribero et al. (29) specifically focused on the involvement of the head and neck region in AD patients. Their findings underscored that the severity of AD in the head and neck region was associated with a significant worsening of QoL (29). Furthermore, the consensus from our study participants indicates that moderate-to-severe AD leads to significant psychological disorders, notably anxiety and mood disorders. This observation is consistent with a study from the Netherlands, which found that adults with AD, particularly those with more severe manifestations, were more prone to experience loneliness and a range of psychiatric disorders (30).

The debilitating nature of AD was another focal point of our study, with a majority of participants categorizing it as both impairing (92.6%) and disabling (81.5%). This sentiment is mirrored in a pan-European study that spanned five countries, revealing that AD patients, even those with milder forms of the disease, reported a heightened burden in terms of medical and psychological comorbidities, overall quality of life, and functional status (31). The consensus also underscored the significant work and productivity impairment in adults with moderate-to-severe AD, a finding that resonates with a Japanese study's outcomes, where AD patients reported elevated overall work and activity impairment (32).

The diagnostic process for atopic dermatitis (AD) remains a topic of ongoing exploration and debate within the dermatological community. Our Delphi method consensus study highlighted several key points in this realm. A significant consensus emerged around the idea that in certain situations, skin biopsies should be considered to exclude other conditions, such as early-stage T-cell cutaneous lymphoma, psoriasis, or dermatitis herpetiformis. This aligns with the findings of Frings et al., which emphasized that histopathology does not reliably differentiate between allergic contact dermatitis, irritant contact dermatitis, and AD, but is instrumental in excluding other conditions like psoriasis, tinea, or T-cell lymphoma (33). Furthermore, our study's consensus underscored that there are currently no validated biomarkers aiding in the diagnosis of AD. This is consistent with the broader literature, as highlighted by a study by Lee, which emphasized the lack of definitive biomarkers for AD, making the establishment of standard diagnostic criteria challenging (34).

The diagnostic criteria for atopic dermatitis (AD) have been a subject of extensive research and discussion within the dermatological community. Our Delphi method consensus study emphasized that the current diagnostic criteria systems are generally deemed sufficient for the diagnosis of AD. This sentiment is supported by a systematic review by Vakharia et al., which analyzed various diagnostic criteria used in AD randomized controlled trials (35). Their findings indicated that while multiple diagnostic criteria exist, there is a convergence toward a set of common features that are consistently used for AD diagnosis across different studies. Moreover, the article by Lee delves into the various diagnostic criteria for AD, highlighting the challenges and the need for reliable diagnostic tools (34). However, it's worth noting that while there's a general consensus on the sufficiency of current diagnostic criteria, the landscape is not without challenges. Flohr pointed out that despite the heightened interest in AD-related research, the multitude of diagnostic criteria and outcome measures can sometimes hamper study comparability (36). This underscores the importance of ongoing efforts to harmonize and standardize diagnostic criteria for AD. Interestingly, our study also shed light on the variability in the time it takes for AD patients to receive a diagnosis from their first symptom. While experts' perspectives varied, the reported average time was <6 months, with some countries like Czechia and Turkiye averaging <4 months, and Russia even <1 month. This variability underscores the need for standardized referral systems with set criteria across different regions.

The assessment of atopic dermatitis (AD) severity is crucial for both clinical practice and research. Our Delphi method consensus study brought forth several significant findings in this domain. A notable consensus was the perspective that AD patients with significantly impaired quality of life can be considered to have moderate-to-severe AD regardless of their Body Surface Area (BSA) involvement. This aligns with the understanding that the physical extent of AD, as measured by BSA, is just one aspect of the disease's impact, and the patient's quality of life is equally, if not more, important (37). Another consensus was the consideration of AD patients with a minimum involvement of 10% BSA as having moderate-to-severe AD. This is in line with the findings from a systematic review by Rehal and Armstrong, which highlighted the use of BSA as a common metric in AD clinical trials (38).

Regarding the scoring systems preferred for estimating AD severity, our study found SCORAD to be the most commonly chosen (74%), followed by EASI (66.6%). This is consistent with the literature, as SCORAD and EASI are among the most frequently used disease-severity instruments in AD clinical trials (38). Interestingly, while experts from Czechia preferred EASI, they found IGA-BSA and SCORAD to be the least common. This regional variation underscores the diverse preferences and practices across different countries. However, it's worth noting that despite the availability of these scoring systems, a significant percentage (88.8%) reported that they are not utilized as much as they should be in daily practice to evaluate AD patients. This highlights a potential gap between research and real-world clinical practice.

The management of atopic dermatitis (AD) is intricate, reflecting the multifaceted nature of the disease. Our Delphi consensus study underscored the variability in treatment approaches, even within the same country, emphasizing the complexity of AD management. This variability is not unique to our study but is echoed in the broader literature, highlighting the challenges faced by clinicians in tailoring treatments to individual patients. For instance, a study by Eichenfield et al. evaluated recent treatment guidelines for AD, emphasizing that while many patients can be managed at the primary care level, the guidelines often cater more to specialists, potentially leading to disparities in treatment approaches (39). This underscores the importance of individualized treatment plans, as each patient's situation is unique, and a one-size-fits-all approach may not be optimal. Another study by Mohan and Lio compared AD management guidelines from different specialty organizations and found notable differences in recommendations, suggesting potential disparities in the perceptions of AD between dermatologists and allergists (40). Such disparities can lead to varied treatment approaches, even within the same country or region. Furthermore, Wollenberg et al. discussed the challenges in managing AD and psoriasis, emphasizing that while our understanding of optimal care plans is increasingly sophisticated, this knowledge is not always reflected in daily clinical practice (27). This gap between theory and practice further accentuates the need for individualized treatment plans. Lastly, Wollenberg et al. highlighted the importance of a holistic approach to AD management, encompassing systemic, topical, and psychological interventions (41). They stressed the need for therapeutic patient education and a multidisciplinary approach, emphasizing that individualized strategies are crucial for ensuring good adherence by both children and their parents.

Despite these differences, there was a strong consensus in our study on several literature-based statements on treatment management such as the proactive treatment approach, involving the use of topical corticosteroids (TCS) or topical calcineurin inhibitors (TCIs) twice a week as well as phototherapy's role as a treatment option for adults with persistent moderate to severe AD, especially before considering systemic agents. A systematic review by Siegfried et al. highlighted the long-term safety of TCS and TCIs in pediatric patients with AD (42). The study found that while TCS has been associated with adverse cutaneous effects like atrophy and increased percutaneous absorption with potential for adverse systemic effects, TCIs, on the other hand, are not associated with these adverse effects even after prolonged use. The study also emphasized that the potential risk of malignancy with TCIs, particularly tacrolimus and pimecrolimus, remains theoretical and has not been conclusively proven. Phototherapy, especially narrowband (NB)-UVB and UVA1, has shown significant beneficial effects against AD. Natural sunlight, broadband (BB)-UVB, UVA, cold-light UVA1, UVAB, full-spectrum light (including UVA, infrared, and visible light), and other forms of phototherapy have been explored for their potential benefits in AD treatment (43). A study by Pavlovsky et al. highlighted the effectiveness of narrowband UVB in pediatric patients with AD, suggesting its potential as a valuable treatment option for this age group (44). Another review by Kemény et al. emphasized the gold standard status of NB-UVB for treating AD, with UVB excimer laser and excimer lamp being particularly effective for localized therapy-resistant lesions (45). Phototherapy is generally considered safe and well-tolerated. However, there are concerns related to prolonged exposure, such as the potential for skin aging, burns, and an increased risk of skin cancer (43). These studies emphasized the long-term safety of phototherapy in pediatric patients with AD, suggesting that while there are potential adverse effects, they are manageable with appropriate precautions (43–45). It is essential to note that while phototherapy is effective, it requires multiple sessions per week, which might not be feasible for all patients. Additionally, it may not be suitable for those with a history of skin cancer or certain photosensitivity disorders.

While the consensus from our Delphi method study highlighted Dupilumab's potential as a first-line systemic treatment for children over 12 years and adults with moderate-to-severe atopic dermatitis (AD) when the disease remains uncontrolled despite optimal topical and/or phototherapies, this statement doesn't reflect nor align with the current literature. The reason for this is that we couldn't include questions on Janus kinase (JAK) inhibitors inhibitors at the time of the study as they were not approved and were not in clinical use. Among the systemic treatments that can be prescribed today, two types of novel agents are attractive and have been approved in many countries to alleviate the symptoms of AD; JAK inhibitors, such as baricitinib (anti-JAK1/2), abrocitinib (anti-JAK1), and upadacitinib (anti-JAK1), and anti-interleukin (IL) signaling antibody, such as dupilumab (anti-IL-4Rα), tralokinumab (anti-IL-13), and nemolizumab (anti-IL-31 Rα). While Dupilumab targets the IL-4 and IL-13 pathways, JAK inhibitors have a broader mechanism of action, targeting multiple cytokines involved in the pathogenesis of AD. This offers a different therapeutic approach and expands the options available for patients with AD, especially those who might not respond optimally to one type of treatment (46). A network meta-analysis evaluating JAK inhibitors, specifically abrocitinib, baricitinib, and upadacitinib, for moderate-to-severe AD revealed that upadacitinib 30 mg may offer improved efficacy, but with a higher incidence of treatment-emergent adverse events in short-term studies. However, abrocitinib 200 mg showed better efficacy relative to other dosages of abrocitinib and baricitinib (47). A similar conclusion was reported by Ducker et al. in another network analysis underlining abrocitinib 200 mg and upadacitinib 30 mg better scores compared to dupilumab (48).

In the evolving landscape of atopic dermatitis (AD) management, our Delphi consensus study has brought to the forefront some pressing concerns and unmet needs. A significant majority of panelists (92.6%) opined that the current topical therapies and immune response modifiers fall short in addressing the therapeutic requirements of patients with moderate-to-severe AD. This sentiment echoes findings from other studies, which have underscored the limitations of existing treatments, particularly in achieving sustained remission and managing severe manifestations of the disease (9). Furthermore, the consensus highlighted the challenges associated with the off-label use of currently available immunosuppressants. A substantial 85.2% of participants concurred on the significant burden of adverse events tied to such off-label use. This is in line with global observations, where concerns about the long-term safety of immunosuppressants have been raised, especially in pediatric populations (49). Perhaps the most striking consensus was the unanimous agreement (100%) on the significant unmet need for innovative topical and systemic agents. The ideal agents would offer prolonged remission and possess a safe side-effect profile, especially for long-term treatment of moderate to severe AD.

In conclusion, while our experts addressed the unmet needs for innovative topical and systemic agents during the study period, JAK inhibitors and IL signaling antibodies have significantly transformed the landscape of AD treatment till the results of this Delphi consensus study is reported on this manuscript. The introduction of JAK inhibitors and IL signaling antibodies has provided patients with an effective alternative to corticosteroids and immunosuppressants, which often come with substantial side effects. Consequently, they have expanded treatment options and shifted the therapeutic paradigm toward more targeted and individualized approaches. However, there is still a need for more long-term safety data on these treatments to fully understand their risk profiles and ensure sustained efficacy and safety for patients over extended periods. Therefore, our consensus study's main outcome—the unmet need for the development and validation of novel therapeutic agents that can fill the existing gaps in AD management—is still present.

## Study limitations

The present study has all the limitations arising from the nature of the Delphi method (23–26). The different representation rates of specialties from dermatology and allergology and limited number of experts from each country result in a limitation in reflecting the approaches and insights at an ideal level. Also new treatments such as JAK inhibitors were not assessed within Delphi rounds as they were not available at the time of the study execution. However, the Delphi method allowed exploring systematically different clinical management approach toward patients with AD, based on the qualified opinion of dermatology and allergology experts in the field.

## Data availability statement

The raw data supporting the conclusions of this article will be made available by the authors, without undue reservation.

## Author contributions

MT: Conceptualization, Formal analysis, Investigation, Methodology, Supervision, Validation, Writing – review & editing. LR: Conceptualization, Formal analysis, Investigation, Methodology, Supervision, Validation, Writing – review & editing. PA: Investigation, Validation, Writing – review & editing. BE: Investigation, Validation, Writing – review & editing. AL'v: Investigation, Validation, Writing – review & editing. SA: Investigation, Validation, Writing – review & editing. EA: Investigation, Validation, Writing – review & editing. NB: Investigation, Validation, Writing – review & editing. SB: Investigation, Validation, Writing – review & editing. MB: Investigation, Validation, Writing – review & editing. MC-O: Investigation, Validation, Writing – review & editing. OE: Investigation, Validation, Writing – review & editing. TE: Investigation, Validation, Writing – review & editing. IE: Investigation, Validation, Writing – review & editing. EF: Investigation, Validation, Writing – review & editing. OF: Investigation, Validation, Writing – review & editing. DF: Investigation, Validation, Writing – review & editing. AG: Investigation, Validation, Writing – review & editing. MK: Investigation, Validation, Writing – review & editing. ALe: Investigation, Validation, Writing – review & editing. AM: Investigation, Validation, Writing – review & editing. NM: Investigation, Validation, Writing – review & editing. WO: Investigation, Validation, Writing – review & editing. EÖ: Investigation, Validation, Writing – review & editing. ZP: Investigation, Validation, Writing – review & editing. AR: Investigation, Validation, Writing – review & editing. MS: Validation, Writing – review & editing, Investigation. BG: Investigation, Validation, Writing – review & editing.
